# Therapeutic Advances in Relapsed and Refractory Peripheral T-Cell Lymphoma

**DOI:** 10.3390/cancers15030589

**Published:** 2023-01-18

**Authors:** Robert Stuver, Alison J. Moskowitz

**Affiliations:** Lymphoma Service, Department of Medicine, Memorial Sloan Kettering Cancer Center, New York, NY 10065, USA

**Keywords:** lymphoma, T-cell lymphoma, T-cell, large-cell, anaplastic

## Abstract

**Simple Summary:**

T-cell lymphomas are a rare, heterogeneous family of lymphomas derived from post-thymic T lymphocytes. For patients with relapsed or refractory disease, outcomes are generally poor, with overall survival usually less than one year in the absence of an allogeneic hematopoietic stem cell transplantation. The historic approach to relapsed or refractory disease has been the use of non-overlapping combination chemotherapy. However, tremendous progress has been made in understanding the pathogenesis of these diseases, leading to a plethora of novel, biologically rational therapies. In this review, we provide an updated evaluation of therapeutic advances in relapsed and refractory T-cell lymphomas, focusing on approved agents and promising investigational regimens with emerging data within the last five years. We provide a basic framework for the management of disease in this setting.

**Abstract:**

Historic outcomes for patients with relapsed or refractory nodal-based T-cell lymphomas are poor, with survival generally measured in months in multiple reports from the late 20th and early 21st century. Until recently, salvage strategies have mostly been borrowed from other aggressive lymphomas. However, dedicated investigations into the pathogenesis of T-cell lymphomas have resulted in an outpouring of therapies that target these diseases in biologically rational strategies. In particular, an evolving appreciation of the multiple complex oncogenic pathways and epigenetic changes that underlie these diseases has led to numerous agents targeting these aberrancies. Moreover, large reports of salvage allogeneic stem cell transplants in T-cell lymphoma have now been published, showing that adaptive immunotherapy is a potentially curative strategy for patients with relapsed or refractory disease. This review highlights therapeutic advances for relapsed or refractory T-cell lymphomas, including cellular therapy and allogeneic stem cell transplant, and provides a framework for management.

## 1. Introduction

The peripheral T-cell lymphomas (PTCL) encompass a broad class of heterogeneous clinicopathologic entities unified in their derivation from a mature, post-thymic T-cell. Altogether, 34 individual neoplasms are recognized in the recently updated 5th edition of the World Health Organization (WHO) Classification of Haematolymphoid Tumours [1]. Similarly, in the contemporaneous International Consensus Classification (ICC) of Mature Lymphoid Neoplasms, 36 unique diseases are described [2]. Despite the sheer number of types and subtypes, T-cell lymphomas are rare diseases, with an annual incidence in the United States of under 2 per 100,000 persons [3]. Individual disease incidence rates vary from 0.4–0.5 per 100,000 for peripheral T-cell lymphoma, not otherwise specified (PTCL-NOS), to ≤0.1 per 100,000 for rarer subtypes, such as hepatosplenic TCL, natural killer/T-cell lymphoma, T-cell prolymphocytic leukemia, and others [3]. This rarity has made the dedicated study and clinical management of T-cell lymphomas exceptionally challenging.

While some histologies have an indolent course, the more common, nodal-based subtypes—PTCL-NOS, angioimmunoblastic TCL (AITL), and anaplastic large cell lymphoma (ALCL)—have a generally more aggressive natural history with worse prognoses (of note, the nomenclature for AITL has slightly changed in the WHO/ICC classification schemas; this review will continue to use AITL for consistency purposes). Even with combination chemotherapy, historic series show 5-year progression- or failure-free survival rates between 20% and 30% for nodal-based diseases (with the exception of anaplastic lymphoma kinase [ALK]-positive ALCL in young patients, which tends to have more favorable outcomes) [4,5,6]. In addition, as many as 30% of patients may be primary refractory to chemotherapy [4,5,6]. Consequently, an unfortunately large group of patients require therapy in the relapsed and refractory (R/R) setting. Thanks to increasingly dedicated investigations into the molecular and genomic landscape of T-cell lymphomas, a plethora of novel, biologically rational therapies are under clinical investigation, and the management of R/R disease is advancing. Herein, we review the outcomes and clinical care of R/R PTCL, with an emphasis on nodal-based diseases and what we consider the most significant recent therapeutic advances.

## 2. Outcomes in Relapsed and Refractory PTCL

Historic outcomes in R/R PTCL have been poor (Table 1).

An early benchmark from the British Columbia Cancer Agency (BCCA) of 153 patients with R/R disease between 1976 and 2010 reported median progression-free survival (PFS) and overall survival (OS) of only 3.1 and 5.5 months, respectively [7]. Importantly, this series reports outcomes in the absence of allogeneic stem cell transplantation (alloSCT). As salvage, most patients received traditional chemotherapy, though nearly one-quarter (24%) of patients received only supportive care. Still, in those who received chemotherapy, outcomes were only marginally improved compared to the whole cohort, with a median PFS and OS of just 3.7 and 6.5 months, respectively. On multivariate analysis of prognostic factors for OS, poor performance status (HR 2.32, 95% CI 1.59–3.37) and progressive disease (as opposed to relapsed) (HR 1.94, 95% CI 1.28–2.96) were associated with worse survival. A similar report from the Modena Cancer Registry of 53 patients with R/R disease between 1997 and 2010 reported a three-year overall survival rate after relapse of just 19% [8]. This historic data highlight the rarity of durable remissions in the R/R setting, especially when treating with chemotherapy alone. 

More recent updates emanate from the International T-cell Lymphoma Project (ITCP) and the Comprehensive Oncology Measures for Peripheral T-cell Lymphoma Treatment Registry (COMPLETE) [9,10]. The ITCP is one of the largest series to date, with over 600 patients with R/R disease across 74 multinational sites between 2006 and 2016 [8]. The median OS in this report was 5.8 months, showing essentially no improvement compared to prior data. In the COMPLETE registry, a prospective North American database of 155 patients with R/R disease between 2010 and 2014, outcomes were noticeably improved, with median OS for patients with relapsed and refractory disease of 29.1 and 12.3 months, respectively [10]. Worse outcomes in the ITCP compared to the COMPLETE registry may be explained by the broad distribution of patients across multiple international sites, higher rates of rare and less favorable histologies, and less availability to investigational agents and alloSCT. Still, the aggregation of these historical datasets reflects the high mortality associated with R/R PTCL.

## 3. Therapeutic Advances

There is no standard approach in treating R/R PTCL (Table 2). 

**Figure 1:**
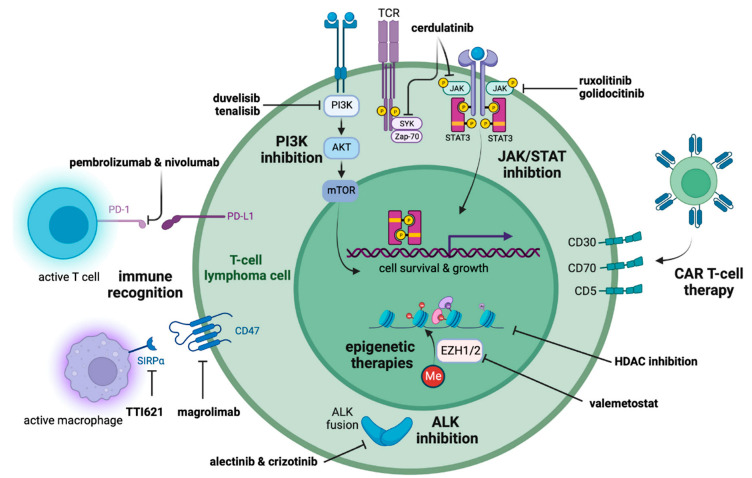
Selected therapeutic advances in peripheral T-cell lymphomas. From top left, clockwise: **PI3K inhibition:** Duvelisib and tenalisib are dual inhibitors of the PI3K/AKT/mTOR pathway through PI3Kγδ inhibition. **JAK/STAT inhibition:** Cerdulatinib is a dual inhibitor of JAK/SYK. Ruxolitinib and golidocitinib are JAK1/2 and JAK1 inhibitors of the JAK/STAT pathway, respectively. **CAR T-cell therapy:** CAR T-cell therapy remains in early study in T-cell lymphoma, with targets including CD30, CD70, CD5, and others. **Epigenetic therapies:** Epigenetic therapies include continued use of HDAC inhibition, as well as exploration of combination therapies using HDAC inhibitors plus other molecules. Valemetostat is an EZH1/2 inhibitor. **ALK inhibition:** Alectinib and crizotinib inhibit ALK fusion proteins in ALK + ALCL. **Immune recognition:** Immune recognition strategies include interruption of the CD47-SIRPα axis and checkpoint blockade in particular histologies (EBV+ T-cell lymphoma, NK/T-cell lymphoma, cutaneous T-cell lymphoma). Abbreviations: ALK, anaplastic lymphoma kinase; CAR, chimeric antigen rector; EZH, enhancer of zeste homolog; HDAC, histone deacetylase inhibitor; JAK, Janus kinase; Me, methyl; mTOR, mammalian target of rapamycin; PD-1, programmed cell death protein 1; PD-L1, programmed death-ligand 1; SIRPa, signal regulatory protein α; STAT, signal transducer and activator of transcription; SYK, spleen tyrosine kinase; TCR, T-cell receptor; Zap-70, Zeta-chain-associated protein kinase-70. Created in BioRender.com.

Historically, a paradigm of second-line combination chemotherapy salvage has been employed [16,17]. However, an increasing appreciation of the complicated pathogenesis of PTCL has led to the development and investigation of rational therapies that attempt to undercut multiple oncogenic mechanisms. Below is a review of these advances followed by a suggested framework for management (Figure 1, Table 3). 

### 3.1. Targeting Oncogenic Pathways

Multiple aberrant cell pathways contribute to the pathogenesis of PTCL, especially those involving T-cell receptor (TCR) and cytokine signaling [18]. TCR signaling requires a highly sophisticated interplay between membrane and intracytosolic proteins to coordinate survival, proliferation, and differentiation responses to external stimuli [18]. Phosphatidylinositol 3-kinase (PI3K), a lipid kinase member of the PI3K/AKT/mammalian target of rapamycin (mTOR) pathway, serves a critical role in T-cell receptor signaling in addition to numerous other immunomodulatory tasks [19]. Targeting this pathway has emerged as an effective therapeutic strategy in PTCL. Duvelisib, an oral inhibitor of PI3K-γδ isoforms, was first explored in a phase I trial of 35 patients with R/R T-cell lymphoma [20]. The objective response rate (ORR) was 50% in the PTCL population, with a CR rate of 19%. Responses were seen across several subtypes, including PTCL-NOS, ALCL, and AITL, as well as rarer histologies, such as subcutaneous panniculitis-like T-cell lymphoma (SPTCL). Phase II evaluation in the PRIMO trial (NCT03372057) has now met its target enrollment. Interim evaluation of 101 patients showed an ORR of 49% with a CR rate of 34%, with notably high responses in AITL (67% ORR) versus ALCL (13% ORR) [21]. Duvelisib is now listed by the National Comprehensive Cancer Center (NCCN) as an option for R/R PTCL. Final results of the PRIMO trial, including subtype-specific responses and the ability to bridge to alloSCT, are eagerly awaited. A second agent, tenalisib, shows similarly promising activity and is being explored in combination regimens (see below) [22]. Additional agents such as copanlisib and linperlisib have also been evaluated as single agents and in combination with chemotherapy [23,24,25]. Recent FDA guidance on PI3K inhibitors in hematologic malignancies (primarily in mature B-cell lymphoproliferative disorders) given concerning survival trends and toxicities leaves the future of PI3K inhibition in T-cell lymphoma somewhat unclear [26].

The Janus kinase (JAK)/signal transducers and activators of transcription (STAT) pathway is a second integral pathway influencing T-cell differentiation and function. Constitutive activation of the JAK/STAT pathway is seen across T-cell lymphomas, resulting from activating *JAK/STAT* mutations as well as other upregulating mechanisms [18]. Inhibiting the JAK/STAT pathway has emerged as a potential strategy in R/R PTCL. Ruxolitinib, a JAK1/2 inhibitor, was evaluated in a biomarker-driven phase II trial in those with R/R PTCL or CTCL [27]. Based on next-generation sequencing for *JAK1, JAK2, STAT3, STAT5,* and immunohistochemistry (IHC) for phosphorylated STAT3 (pSTAT3), patients were enrolled into one of three cohorts: (1) activating *JAK* and/or *STAT* mutation, (2) no *JAK/STAT* mutation but ≥30% pSTAT3 expression by IHC, or (3) neither. The ORR among 48 patients was 23%, with the ORR by cohorts 1, 2, and 3 being 28%, 31%, and 12%, respectively (*p* = 0.20 for cohorts 1 and 2 versus 3). The clinical benefit rate (CBR), which captures response and durability, varied by cohort as well, with CBRs for cohorts 1, 2, and 3 of 48%, 36%, and 18% (*p* = 0.073). Subtypes enriched for *JAK/STAT* mutations, such as T-prolymphocytic leukemia (T-PLL) and T-large granular lymphocytic leukemia (T-LGL), notably achieved relatively higher ORR and CBR [28,29,30]. Suggestions that overactive PI3K/AKT/mTOR signaling may confer resistance have prompted evaluations of duvelisib plus ruxolitinib in an ongoing phase I trial (NCT05010005). Additionally, an increasing appreciation of *JAK* fusion events in T-cell lymphomas [31,32,33,34] prompted an ongoing rare subtype expansion cohort of ruxolitinib in T-PLL, T-LGL, and non-mycoses fungoides cutaneous T-cell lymphoma (CTCL) (NCT02974647).

Other investigative agents targeting this pathway include cerdulatinib, a dual inhibitor of JAK and spleen tyrosine kinase (SYK), which is overexpressed in a majority of PTCL [46]. In a phase II trial of R/R PTCL and CTCL, cerdulatinib showed an ORR of 35% in the PTCL cohort and 35% in the CTCL cohort [47]. Responses were notably high in AITL and other T-cell lymphomas with a T-follicular helper (TFH) cell phenotype, with an ORR of 55% and a CR rate of 41%. In those with CTCL, certain patients achieved a rapid improvement in pruritis independent of tumor response, which may translate to an overall improvement in symptom burden. Golidocitinib, a selective JAK1 inhibitor, has been granted Fast Track Designation by the Food and Drug Administration for R/R PTCL and is being evaluated in an ongoing evaluation in a phase I/II trial (NCT04105010), with encouraging response rates thus far [37]. Full exploitation of the JAK/STAT pathway in biomarker-informed paradigms is a promising strategy in R/R PTCL.

### 3.2. Altering the Epigenome

The last decade has witnessed the realization of a group of nodal T-cell lymphomas that bear the immunophenotype and gene expression profiling of TFH cells, the prototype being AITL. Other members of this disease family include nodal PTCL with a TFH phenotype and follicular T-cell lymphoma (both of which have slightly varied names in the WHO/ICC classification schemas). Molecular characterizations show that these lymphomas share mutational and gene expression profiling characterized by aberrations in epigenetic functioning, especially in DNA and histone methylation. Highly recurrent mutations include *TET2* and *DNMT3A*, as well as *RHOA*^G17V^, *IDH2*^R172^, and mutations affecting TCR signaling [48,49,50].

Importantly, these findings have translated into the clinical realization that these diseases are sensitive to epigenetic therapies. Belinostat, a histone deacetylase (HDAC) inhibitor, was approved by the FDA for use in R/R PTCL in 2014 [11]. Romidepsin, a second HDAC inhibitor, was previously approved in 2011 for R/R PTCL but has since had its accelerated approval voluntarily withdrawn based on a negative phase III trial in upfront PTCL, though the agent is still available for use [15]. A third HDAC inhibitor, vorinostat, is approved for use in CTCL. While not particularly apparent in the original phase II investigations, later (and larger) analyses have shown that these agents appear to have the greatest effect in TFH lymphomas.

For example, in a multi-institutional retrospective effort of 127 patients with R/R nodal T-cell lymphoma treated with an HDAC inhibitor, patients with a TFH phenotype had an improved ORR of 57% versus 30% in those with a non-TFH phenotype (*p* = 0.004) [51]. In multivariate analyses for ORR, a TFH phenotype was significantly associated with response to HDAC inhibition (*p* = 0.009). Mutational signatures typical of the TFH (*TET2*, *DNMT3A*, and *RHOA*) phenotype were significantly more common in responders than non-responders. Accounting for goals of therapy, side effect profiles, and patient preference, HDAC inhibitors may be prioritized over other therapies in patients with R/R TFH lymphomas, though no prospective comparisons exist. Combinations involving romidepsin have been investigated as well. Most encouraging might be romidepsin plus PI3K inhibition. Romidepsin plus duvelisib was evaluated in a phase I dose expansion trial in R/R PTCL, resulting in an ORR of 56% (44% CR rate) and allowing for bridging to potentially curative alloSCT [40]. As expected, responses were highest in AITL (71% ORR). Exploratory mutational analyses showed associations with *TET2*, *LOF*, *RHOA*, and *VAV1* mutations and response, whereas *TP53* mutations were exclusively seen in non-responders. Similarly, in a smaller trial of romidepsin plus tenalisib (an inhibitor of PI3K-γδ and salt-inducible kinase 3 [SIK3]), high response rates were observed in PTCL (75% ORR) and CTCL (35% ORR) [41]. Other combinations with published efficacy data include romidepsin plus lenalidomide, romidepsin plus azacytidine, and romidepsin plus lenalidomide plus carfilzomib [52,53]. No combination regimen is clearly superior to another, and none is currently approved for use.

Similarly, hypomethylating agents have been explored in T-cell lymphomas with a TFH phenotype given the known efficacy of this mechanism in myeloid disorders enriched for mutations in *TET2* and *DNMT3A*, mutations that are similarly frequent in T-cell lymphomas with a TFH phenotype [54,55,56,57]. In a small retrospective series of 12 patients with AITL, nine patients demonstrated a response [58]. This report is notable in that five patients had a concomitant myeloid disorder. These findings led to the recently presented randomized phase III ORACLE study (NCT03593018) of oral azacytidine versus investigator’s choice in R/R AITL and other T-cell lymphomas with a TFH phenotype [59]. The study included 86 patients and was powered to show a median PFS benefit of 12 months in the oral azacytidine arm versus five months in the standard arm. The primary PFS endpoint was not met, with a median PFS of 5.6 months versus 2.8 months for those treated with oral azacytidine versus investigator’s choice, respectively (HR 0.634, 95% CI 0.38–1.07). The best overall response at three months in the oral azacytidine arm was surprisingly less than in the investigator’s choice arm, at 33.3% (95% CI, 19.6–49.5%) versus 43.2% (95% CI, 28.3–59.0%). Mutations in *TET2*, *RHOA*, and *IDH2* unexpectedly did not associate with survival. These disappointing results show that prospective comparisons, while logistically challenging, are important in evaluating drug efficacy in T-cell lymphomas. Combination strategies of azacytidine may still have merit, and an upfront, randomized trial of azacytidine-CHO(E)P versus duvelisib-CHO(E)P versus CHO(E)P is ongoing (NCT04803201).

Finally, the results of VALENTINE-PTCL01, a phase II registration trial of valemetostat, are pending. Valemetostat is a histone methyltransferase inhibitor—specifically, an inhibitor of enhancer of zeste homolog 1 (EZH1) and EZH2. EZH2 is widely overexpressed in T-cell lymphomas, and EZH1/2 inhibitors are promising therapies [60,61]. In a dedicated phase II study (NCT014102150) in Japan for patients with R/R ATL, a high ORR of 48% was observed in 25 patients, 24 of whom had been previously treated with mogamulizumab [62]. In a separate phase I trial of valemetostat in all R/R non-Hodgkin lymphomas, including PTCL, an ORR of 56% was seen in PTCL [45]. Responses were notably high in AITL (71% ORR). These results have prompted the global VALENTINE-PTCL01 trial, which recently completed accrual. 

### 3.3. Harnessing the Immune System

Exploiting the immune system has revolutionized the treatment of numerous solid and hematologic malignancies, though has proved challenging in T-cell lymphomas. This is largely owing to the complexities of attempting to deplete malignant T cells while simultaneously attempting to unleash an anti-tumor T-cell response [63]. The inhibitory receptor programmed cell death protein 1 (PD-1) and its ligand, programmed death-ligand 1 (PD-L1), are widely expressed by malignant T-cell lymphomas and surrounding non-malignant T cells [64,65]. However, the mere presence of these markers needs to be interpreted cautiously. Preclinical models suggest PD-1 functions as a haploinsufficient tumor suppressor of T-cell lymphoma pathogenesis, and therefore checkpoint inhibitors have the potential to accelerate existing T-cell lymphoma or reactivate T cell clones and paradoxically promote tumorigenesis [66]. These concerns were borne out in a phase II trial of nivolumab in adult T-cell leukemia/lymphoma (ATLL), in which the first three patients experienced rapid progression after a single infusion [67,68]. However, a similar trial of nivolumab in ATLL patients in Japan did not observe hyperprogression [69]. Elsewhere, a phase II trial of single-agent pembrolizumab in R/R T-cell lymphomas was halted early after a preplanned interim futility analysis based on PFS, despite a modest overall response rate of 33% and rare durable responses [39]. A similar evaluation of a phase II trial of nivolumab in R/R PTCL resulted in four cases of hyperprogression [70]. Nivolumab plus romidepsin in R/R PTCL showed modest efficacy, though hyperprogression was still observed [71]. Tislelizumab, an investigational anti-PD1 monoclonal antibody designed to minimize antibody-dependent macrophage-mediated killing of T effector cells, showed modest results without hyperprogression in a recently reported multinational phase II trial [72]. Clearly, these findings give overall pause to investigators, and neither pembrolizumab nor nivolumab are available for use outside of a clinical trial. 

The most encouraging results of checkpoint blockade in T-cell lymphomas appear to be in histologies associated with Epstein–Barr virus (EBV) and CTCL. In seven patients with R/R extranodal natural killer (NK)/T-cell lymphoma who failed L-asparaginase regimens, salvage pembrolizumab resulted in an ORR of 100% [73]. At the median follow-up of six months, one patient had died of disease, but all others remained alive, and five patients remained free of disease. Case reports of nivolumab have shown similarly high efficacy [74]. Both pembrolizumab and nivolumab are now listed by the NCCN as preferred regimens for treating R/R extranodal NK/T-cell lymphoma. In CTCL, a phase II trial of pembrolizumab in 24 patients with R/R mycoses fungoides and Sezary syndrome showed an ORR of 38% [75]. In those who responded, duration of response (DOR) was sustained, with median DOR not reached after a median follow-up of 58 weeks. Only one patient lost response, and that patient had discontinued treatment because of treatment-related pneumonitis. While no patients experienced hyperprogression, eight patients did experience a marked worsening of erythema and pruritis, though symptoms mostly remitted within 12 weeks and none required treatment discontinuation. PD-L1 structural variants, which can be seen in large cell transformation of mycoses fungoides, may allow sensitivity to checkpoint blockade and could prompt consideration for pembrolizumab use [76]. Pembrolizumab is not approved for CTCL but has NCCN compendium listing and can be used off-label. These results demonstrate that checkpoint blockade has merit in T-cell lymphomas, but a deeper understanding of the role of PD-1/PD-L1 in tumorigenesis and immune evasion is needed to safely continue evaluation. 

An additional checkpoint that has garnered interest is CD47, a transmembrane protein member of the immunoglobulin family that inhibits cellular phagocytosis through its interaction with signal receptor protein-alpha (SIRPɑ), a protein receptor expressed on phagocytic cells, such as macrophages [77]. As such, CD47 is commonly thought of as a “do not eat me” signal, and it is overly expressed in numerous malignancies, including non-Hodgkin lymphomas [77]. Multiple anti-CD47 compounds that block this signal and allow immune recognition and clearance are under investigation. TTI-621 (SIRPɑ-IgG Fc), a soluble fusion protein that binds to CD47, shows activity in PTCL and CTCL as monotherapy [42,43]. Magrolimab, a first-in-class anti-CD47 antibody, has efficacy in B-cell lymphoma [78], and is being evaluated in combination with mogamulizumab for R/R T-cell lymphomas in a phase I/II study (NCT04541017). Of note, this study (and others involving magrolimab) was briefly suspended in 2022 over concerns of unexpected serious side effects in an unrelated trial of magrolimab and azacytidine in myelodysplastic syndrome. Neither magrolimab nor TTI-621 are available outside of clinical trials. 

Other agents include lenalidomide, an immunomodulatory agent, and mogamulizumab, an anti-CCR4 antibody [79,80,81]. An early evaluation of 10 patients with PTCL-NOS treated with lenalidomide showed encouraging single-agent activity, with an ORR of 30% [79]. In a larger, phase II trial of 54 patients with R/R T-cell lymphoma, lenalidomide monotherapy displayed an ORR of 22%, with a CR rate of 11% [80]. Finally, similar response rates were observed in a phase II trial of lenalidomide monotherapy in patients with R/R T-cell lymphoma and patients with untreated T-cell lymphoma who were not candidates for combination chemotherapy [81]. Single-agent lenalidomide has an NCCN compendium listing for palliative intent therapy in untreated nodal PTCL or as subsequent therapy in R/R disease. Mogamulizumab, an anti-CCR4 antibody, is FDA-approved for R/R CTCL and in Japan for R/R ATL [82,83,84,85]. However, mogamulizumab was evaluated in a phase II trial of patients with R/R CCR4-positive PTCL-NOS, AITL, and ALCL, and transformed mycoses fungoides and resulted in an ORR of 11%; similarly, in a phase II trial in patients with R/R ATL versus investigator’s choice, mogamulizumab resulted in an ORR of 11% [83,84]. Mogamulizumab is not approved in the United States for nodal PTCL or ATLL. 

Finally, cellular therapies remain in infancy in T-cell lymphomas. Results of an ongoing study (NCT0450246) of allogeneic CD70-targeting chimeric antigen receptor T-cell (CAR T-cell) therapy have recently been reported [36]. Patients with R/R PTCL and CTCL were treated at various dose levels following fludarabine plus cyclophosphamide lymphodepletion. In an interim analysis of 15 patients, the ORR at dose-level 3 was 71% (29% CR). No dose-limiting toxicities, ≥grade 3 cytokine release syndrome, or ≥grade 3 immune effector cell-associated neurotoxicity syndrome were observed. Dose expansion is ongoing. An autologous CD5 CAR T-cell therapy was evaluated in a phase I trial of nine patients with R/R PTCL; responses were observed in four patients, allowing three to proceed to alloSCT [86]. Cytopenias were observed in all patients but mostly recovered within 28 days, and no severe infections occurred. Other targets, including CD30, have been explored to a lesser extent [87,88,89]. A registration-directed phase II trial of AFM13, a CD30/CD16A bispecific antibody, in CD30-positive PTCL or transformed mycoses fungoides, has completed enrollment (NCT04101331) [35]. While promising, the role of cellular therapy in T-cell lymphomas is unclear and depends on further investigation. 

## 4. Other Therapeutic Advances

### 4.1. ALK Inhibition

ALK-positive ALCL is defined by the constitutive activation of ALK, a receptor tyrosine kinase that is normally only expressed in the nervous system. Chromosomal translocations, such as t(2;5) (p23;q35), lead to gene fusion events that result in the oncogenic activation of ALK [13]. Accordingly, ALK inhibition has emerged as an important strategy in R/R ALK-positive ALCL. Crizotinib, a first generation ALK inhibitor, is now FDA-approved for patients ≤ 21 years of age with R/R ALK-positive ALCL. Efficacy is seen in adults as well, as evidenced in a phase II trial of crizotinib in 12 patients with R/R ALK-positive ALCL, in which the ORR was 84% with a 59% CR rate [90]. Alectinib, a second-generation ALK inhibitor, is approved for R/R ALK-positive ALCL in Japan based on a phase II trial that showed similarly high response rates [91]. Both crizotinib and alectinib are now listed by the NCCN as second-line therapies in R/R ALK-positive ALCL. Of note, alectinib has central nervous system (CNS) penetration and has shown activity in patients with CNS involvement [92,93]. Lorlatinib, a third generation ALK inhibitor, is being evaluated in patients who have failed chemotherapy and a prior ALK inhibitor (NCT03505554).

### 4.2. Targeting EBV

In EBV-positive lymphomas, including T-cell lymphomas such as extranodal NK/T-cell lymphoma and systemic chronic active EBV disease, the combination of nanatinostat, a class I selective HDAC inhibitor, and valganciclovir shows promising efficacy [38]. In an exploratory study of 55 patients with EBV-positive lymphoma, this combination resulted in an ORR and CR rate of 40% and 19%, respectively. In T-cell lymphomas, the ORR was higher at 60%. Activity was seen in other lymphomas as well, such as AITL, in which EBV is characteristically positive in non-malignant cells within the tumor microenvironment. This combination is being explored in a phase II trial (NCT05011058). 

## 5. Allogeneic Transplant

While the above therapies are critically important advances in the treatment of R/R T-cell lymphomas, none are reliably curative. Long-term responders are indeed seen, but the duration of response is generally less than desired, and there are no data as to when these therapies can be stopped in those who have achieved a disease-free state. Therefore, the role of alloSCT has gained increasing attention as the one modality that has been shown to achieve durable, potentially curative remissions in large numbers of patients. Recently, a very large retrospective report of alloSCT outcomes of 1942 patients was performed through collaboration between the European Society for Blood and Marrow Transplantation (ESBMT) and the Center for International Blood and Marrow Transplant Research (CIBMTR) [94]. This study included only patients with PTCL-NOS, AITL, and ALCL and stratified patients by donor source, reporting 3-year PFS and OS of 48–52% and 60–64%, respectively (depending on donor source). There were no statistically significant survival differences by donor source. The 3-year non-relapse mortality rate varied from 21–24%, and the 3-year cumulative incidence of relapse varied from 25–29%. In a similarly large series of 508 patients with all subtypes of T-cell lymphomas across 12 institutions, the 5-year PFS and OS rates were 40% and 51%, respectively [95]. In comparison to historical data, no other therapeutic modality can offer this degree of prolonged survival, and the stark reality remains that without alloSCT, most patients with R/R disease die of lymphoma [9]. In these series, in addition to many others, a unifying finding is that disease status at the time of transplant is a critical prognostic factor. In the series of 508 patients, the median PFS for those in CR at transplant was significantly greater than those in PR (44.6 months vs. 8.6 months; *p* < 0.001) [95]. For those with progressive disease, PFS was a mere 3.5 months. Similarly, on multivariate analyses in the ESBMT/CIBMTR series, disease status was significantly associated with PFS, OS, and relapse incidence [94]. Additional factors associated with significantly improved PFS included age <40, Karnofsky performance status ≥ 90, and AITL histology (versus PTCL and ALCL). These recent reports show that alloSCT in remission for those with R/R disease can be a curative strategy. 

The role of autologous stem cell transplantation (autoSCT) in R/R disease is unclear and has only been evaluated retrospectively. Historically, a paradigm of second-line combination chemotherapy regimens was used, with regimens such as ICE (ifosphamide, carboplatin, and etoposide) or DHAP (dexamethasone, cytarabine, carboplatin, and etoposide) [16,17]. Borrowing from the PARMA trial in diffuse-large B-cell lymphoma [96], HDT/ASCR has been attempted as well for those in remission in the R/R setting [97,98,99,100,101,102,103,104]. Results are variable and challenging to interpret in aggregate, though general conclusions from societies such as the NCCN and British Society for Haematology are that HDT/ASCR less frequently results in durable benefit in patients with R/R disease as compared to alloSCT [105,106]. The Memorial Sloan Kettering Cancer Center experience of salvage ICE (ifosphamide, carboplatin, and etoposide) followed by autoSCT showed few durable responses with a median PFS of 6 months [16]. However, in patients with chemosensitive disease, especially those with ALCL, autoSCT has been shown to be effective and associated with lower non-relapse mortality than alloSCT [101]. A prospective evaluation would be welcomed. 

## 6. Framework in Management

While the above discussion highlights the immense progress made in treating R/R PTCL in the last several years, there remains no standard approach to management. Noting the potentially curative outcomes with alloSCT detailed above, the framework for managing patients with R/R PTCL largely hinges upon if the goal of therapy is to bridge to alloSCT. A prior suggested framework based on three groups has been described [107]. The first group (“transplant soon”) are those eligible for alloSCT and for whom a donor has been identified. The second group (“transplant never”) are those who are either ineligible or have decided not to pursue alloSCT. The third (“transplant unclear”) encompasses most patients, being a group in which eligibility and donor status are unclear. While this framework still has merit, obstacles surrounding donor options for alloSCT are thankfully less frequent given the increasing use and similar outcomes with haploidentical donors [87,108,109]. In our practice, we refer nearly all patients with R/R disease to a transplant specialist. 

In addition, this framework, devised nearly ten years ago, suggested the use of combination chemotherapy for those in the “transplant soon” group in attempts to achieve a quick, deep response. However, thanks to the many novel options described above, the choice between salvage chemotherapy and non-chemotherapeutic options is less clear. Importantly, no prospective comparisons between salvage chemotherapy and non-chemotherapy agents exist, though some observational reports show improved response rates and survival in those with R/R disease treated with non-chemotherapeutic drugs [110,111]. Our choice of salvage therapy depends primarily on prior therapies, disease histology, and clinical trial eligibility. We favor clinical trial enrollment for most patients. In those with TFH histology, such as AITL, we favor the use of epigenetic therapies, and in ALK-positive ALCL, we prioritize the use of ALK inhibition. While mutational sequencing is increasingly utilized in the workup of T-cell lymphomas, management based on identified abnormalities is not routine. Evaluation of biomarker-informed strategies based on mutational profiles and other surrogates for underlying biology will be paramount to refining treatment paradigms in the coming years and should be accomplished through thoughtful clinical trial design [112]. Our current approach to treatment is described elsewhere [44].

## 7. Conclusions

The clinical management of R/R T-cell lymphomas is challenging owing to the rarity and heterogeneity of these diseases, historically forcing clinicians to rely on salvage therapy borrowed from other aggressive lymphoma subtypes. Thanks to multiple dedicated molecular investigations into the pathogenesis of T-cell lymphomas, numerous rational agents are under exploration, and therapeutic advances are occurring. Particularly promising are strategies to exploit oncogenic pathways, such as the PI3K/AKT/mTOR and JAK/STAT pathways, with agents such as duvelisib and ruxolitinib. In addition, an increasing recognition of the various epigenetic abnormalities that are seen in TFH lymphomas has led to numerous promising efforts evaluating epigenetic therapies, such as valemetostat and rational combinations of agents with romidepsin. Cellular therapies are at the outset in T-cell lymphomas, but the first large report of an allogeneic CD70 CAR T-cell therapy hints at promise in this modality. Finally, recent large reports of alloSCT outcomes show that alloSCT is a potentially curative strategy in R/R T-cell lymphoma. Strategies to facilitate bridging to alloSCT are the goal in fit patients. In the coming years, we anticipate continued therapeutic advances and further refinement of treatment paradigms based on histology, mutations, and other surrogates of biology.

## Figures and Tables

**Table 1:** Historic Outcomes in relapsed/refractory T-cell lymphomas.

Series	Years	Patient Number	PFS (m)	OS (m)
BCCA	1976–2010	153	3.1	5.5
Modena	1997–2010	53	NR	2.5
ITCP	2006–2016	633	NR	5.8
COMPLETE	2010–2014	155	9.6	relapsed: 29.1 refractory: 12.3

BCCA, British Columbia Cancer Agency; COMPLETE, Comprehensive Oncology Measures for Peripheral T-cell Lymphoma; ITCP, International T-cell Lymphoma Project; m, months; NR, not reported.

**Table 2:** Currently approved therapies for relapsed/refractory nodal peripheral T-cell lymphomas.

Agent	Approval	Mechanism	Response	Median DOR (m)
Belinostat [11]	R/R PTCL	HDACi	ORR: 25.8% CR: 10.8%	13.6
Brentuximab vedotin [12]	R/R systemic ALCL after 1 prior multi-agent chemotherapy regimen	CD30 ADC linked to MMAE	ALCL: ORR: 86% CR: 57% PTCL/AITL: ORR: 41% CR: 24%	ALCL: 25.6 PTCL/AITL: 7.6
Crizotinib [13]	R/R ALK + ALCL in pediatric patients ≥1 year and young adults	ALK inhibitor	ORR: 83.3% CR: 58.3%	39
Pralatrexate [14]	R/R PTCL	DHFRi	ORR: 29% CR: 10%	10
Romidepsin [15]	withdrawn for PTCL in 2021 (still available for use)	HDACi	ORR: 25–38% CR: 15–18%	8.9–17

ADC, antibody drug conjugate; AITL, angioimmunoblastic T-cell lymphoma; ALK, anaplastic lymphoma kinase; ALCL, anaplastic large cell lymphoma; CR, complete response; DHFRi, dihydrofolate reductase inhibitor; DOR, duration of response; HDACi, histone deacetylase inhibitor; MMAE, monomethyl auristatin E; ORR, objective response rate; PTCL, peripheral T-cell lymphoma; R/R, relapsed/refractory.

**Table 3:** Selected agents/regimens under investigation in relapsed/refractory T-cell lymphomas.

Agent	Trial (Phase)	Mechanism	Response	Median DOR (m)	Notes
AFM13 [35]	NCT04101331 (II)	CD16A/CD30 bispecific	NR	NR	Phase II registration results pending
CTX130 [36]	NCT04502446 (I)	Anti-CD70 allo CAR T-cell	ORR: 70% CR: 30% (at DL ≥ 3)	NR	Dose expansion ongoing
Duvelisib [20]	NCT03372057 (II)	PI3K-γδ inhibitor	ORR: 49% CR: 34%	7.7	Full phase II results pending
Golidocitinib [37]	NCT04105010 (I/II)	JAK1 inhibitor	ORR: 43% CR: 22%	NR	--
Nanatinostat + valganciclovir [38]	NCT03397706 (I/II)	HDACi + anti-viral	ORR: 40% CR: 19%	10.4	EBV+ lymphomas
Pembrolizumab [39]	NCT03021057 (II)	Anti-PD-1 Ab	ORR: 100% CR: not reported	NR	R/R NK/T-cell lymphomas
Romidepsin + duvelisib [40]	NCT02783625 (I)	HDACi + PI3K-γδ inhibitor	ORR: 56% CR: 44%	NR	*TET2*, *LOF*, *RHOA*, *VAV1* mts assoc. w/response
Romidepsin + tenalisib [41]	NCT03770000 (I/II)	HDACi + PI3K-γδ/SIK3 inhibitor	ORR: 63% CR: 26%	5.0	--
TTI621 [42,43]	NCT02663518 (I)	SIRPα-IgG Fc	ORR: 25% CR: 3%	5.9 (median treatment duration)	--
Tenalisib [21]	NCT02567656 (I)	PI3K-γδ inhibitor	ORR: 46% CR: 9%	4.9	--
Ruxolitinib [44]	NCT02974647 (II)	JAK 1/2 inhibitor	ORR: 25% CR: 6%	8.4	Differential response seen by *JAK/STAT* mts/pSTAT3
Valemetostat [45]	NCT02732275 (I)	EZH1/2 inhibitor	ORR: 55.6% CR: 24%	12.9	Phase II registration trial completed accrual

Ab, antibody; CAR, chimeric antigen receptor; CCR4, C-C chemokine receptor 4; CR, complete response; CTCL, cutaneous T-cell lymphoma; DL, dose level; DOR, duration of response; EBV, Epstein–Barr virus; EZH, enhancer of zeste homolog; HDACi, histone deacetylase inhibitor; IgG, immunoglobulin; JAK, Janus kinase; mts, mutations; NK, natural killer; NR, not reported; ORR, objective response rate; PD-1, programmed cell death protein 1; ph, phase; PI3K, phosphoinositide 3-kinase; PTCL-NOS, peripheral T-cell lymphoma, not otherwise specified; SIRPα, signal regulatory protein α; SIK, serine/threonine kinase; STAT, signal transducer and activator of transcription; SYK, spleen tyrosine kinase.
